# The influence of nutritional marketing on the preferences, attitudes and consumption of children from 6 to 10 years old

**DOI:** 10.1093/eurpub/ckac129.513

**Published:** 2022-10-25

**Authors:** J Lima, B Ferreira, J Figueiredo

**Affiliations:** 1 Polytechnic Institute of Coimbra, ESTeSC - Coimbra Health School, Coimbra, Portugal; 2 ciTechCare, Center for Innovative Care and Health Technology, Porto, Portugal; 3 GreenUPorto, Sustainable Agrifood Production, Porto, Portugal

## Abstract

**Introduction:**

Childhood obesity is a natural response to the modern food environment. A daily consumption of sugar that exceeds the WHO recommendation by 40.7% of Portuguese children influence this condition. It is recognized that children spend more time watching television and youtube, absorbing commercials about food, being susceptible to these messages and negative health literacy. Nutrition marketing is involved in these ads and most of them promote foods high in fat, sugar and salt through persuasive techniques.

**Purpose:**

Assess implications of exposure to nutritional marketing through television and YouTube on preferences, attitudes and consumption of children from 6 to 10 years old and contribute to the increment of health literacy.

**Materials and methods:**

Data collection through two online questionnaires about television and youtube habits and children's food preferences, developed by the research team. Statistical analysis was performed using IBM SPSS Statistics software.

**Results:**

Forty-two students who were attending a private school, aged between 6 and 10 were evaluated. Access to youtube has led to less healthy choices for breakfast in 95% of students (p = 0.038). It was found that most children do not have TV in their room and have a more balanced breakfast (81%). Cereals, soft drinks and biscuits are foods groups that children see the most in the tv commercials (p = 0.001).

**Conclusions:**

Exposure to nutritional marketing influences children's preferences, consumption and preferences. An active strategy should be encourage to increment health literacy levels at the family level influencing therefore children's healthier choices.

